# Primary Negative Prognostic Factors in Pediatric and Adult Patients Undergoing Trigger Finger Surgery

**DOI:** 10.7759/cureus.55281

**Published:** 2024-02-29

**Authors:** Muhammed Köroğlu, Mustafa Karakaplan, Mustafa Yıldız, Mehmet Eren, Emre Ergen, İpek Balıkçı Çiçek, Okan Aslantürk, Kadir Ertem

**Affiliations:** 1 Orthopaedics and Traumatology, İnönü University School of Medicine, Malatya, TUR; 2 Biostatistics and Epidemiology, İnönü University School of Medicine, Malatya, TUR; 3 Orthopaedics and Traumatology, İnönü University Medical School, Malatya, Malatya, TUR

**Keywords:** pediatric, patient prognosis, duration of symptoms, green classification, a1 pulley release, trigger finger

## Abstract

Objectives

This study aims to investigate the negative prognostic indicators of pediatric and adult trigger finger surgery patients concerning complications, recurrence, and satisfaction.

Methods

A retrospective study was conducted on 61 patients with a total of 91 trigger fingers, including 31 in children and 30 in adult patients, all of whom were treated using a standardized surgical technique. The study considered several demographic and clinical factors, including age, gender, dominant hand, body mass index, occupation, history of trauma, single or multiple finger involvement, staging according to Green classification, diabetes mellitus, comorbidities, recurrence, revision surgery, utilization of non-surgical treatment methods, need for rehabilitation after surgery, time to return to work, the time interval from clinic initiation to the surgery, satisfaction and the duration of the follow-up period. In addition, the quick version of the disabilities of the arm, shoulder, and hand (QDASH); and the visual analog scale (VAS) were used to assess patients’ data.

Results

In adult patients, a statistically significant relationship was observed between the increasing grade of the Green stage and complication rate (p<0.001), recurrence (p<0.001), and lower satisfaction (p<0.001). No statistically significant relationship was identified between Green's classification and complications (p=0.129), recurrence (p=0.854), or satisfaction (p=0.143) in pediatric patients. While a statistically significant relationship existed between the time interval from clinic initiation to surgery and complications (p=0.033) in adult patients, no significant relationships were observed for recurrence or satisfaction. Conversely, there was no statistically significant relationship between the time interval from clinic initiation to surgery and complications, recurrence, or satisfaction in pediatric patients.

Conclusion

This study demonstrates that increasing the grade of the Green stage and duration of symptoms before surgery were the substantial factors contributing to prognosis in adult patients but not in pediatric patients. These findings can assist physicians during patients’ treatment management. We suggest that physicians consider these factors for patients’ satisfaction.

## Introduction

Trigger finger, medically recognized as stenosing tenosynovitis, is a prevalent etiological factor contributing to hand disorders [1]. The manifestation of the trigger finger exhibits a bimodal distribution in its incidence, characterized by an initial peak before the age of eight, followed by a secondary peak observed in individuals within the age range of their 40s and 50s [2]. Predominantly, the prevalence of trigger fingers is more pronounced among the adult population [3]. In cases where the trigger finger afflicts children, it demonstrates an equal predilection for both genders and primarily manifests in the digit of the thumb. Conversely, females exhibit a significantly higher susceptibility to trigger finger among adults, notably in their dominant hand [3]. The manifestation of the trigger finger, a source of considerable distress for patients, arises from an incongruity between the volume of the flexor tendon sheath and the dimensions of its enclosed contents [4]. Patients frequently describe concerns regarding digit snapping, catching, or locking, concurrently reporting tenderness over the A1 pulley. Critical indicators of the trigger finger include a limited range of motion (ROM), notably characterized by an extension deficit in the proximal interphalangeal joint, and an inability to achieve complete flexion of the affected digit [5]. Underlying etiologies such as diabetes mellitus, comorbidity with hand disorders, tendon injuries, and tumors should be taken into consideration when making the diagnosis in patients [6,7,8]. In addition, anatomic anomalies and underlying conditions should be considered in pediatric patients [9]. Non-surgical treatments can be applied to trigger finger patients and surgical treatment modalities such as percutaneous, mini-open, or open A1 pulley release [10,11]. This study aims to investigate the negative prognostic indicators of pediatric and adult trigger finger surgery patients concerning complications, recurrence, and satisfaction. The hypothesis of the study suggests that delayed surgical intervention time and advanced Green classification adversely affect the results of trigger finger surgery in adults. However, these factors do not affect the clinical results in pediatric patients.

## Materials and methods

Between April 2014 and September 2022, our clinic performed surgical releases on 121 patients diagnosed with trigger finger. A retrospective study was conducted on 61 patients with a total of 91 trigger fingers, including 31 in children and 30 in adult patients, all of whom were treated using a uniform open surgical method (Table 1).

**Table 1:** Baseline characteristics of the study sample

VARIABLES		All patients [n (%)]	Adult [n (%)]	Child [n (%)]
Age	Child	31 (50.82)	-	-
	Adult	30 (49.18)		
Gender	Male	22 (36.07)	12 (40.00)	10 (32.26)
	Female	39 (63.93)	18 (60.00)	21 (67.74)
Number of affected fingers	Single	35 (57.38)	15 (50.00)	20 (64.52)
	Multiple	26 (42.62)	15 (50.00)	11 (35.48)
Grade- Green	2	6 (10.00)	4 (13.79)	2 (6.45)
	3	49 (81.67)	22 (75.86)	27 (87.10)
	4	5 (8.33)	3 (10.34)	2 (6.45)
Comorbidity	Yes	23 (37.70)	20 (66.67)	3 (9.68)
	No	38 (62.30)	10 (33.33)	28 (90.32)
Diabetes	Yes	12 (19.67)	12 (40.00)	0 (0.00)
	No	49 (80.33)	18 (60.00)	31 (100.00)
Additional operation	Yes	3 (4.92)	3 (10.00)	0 (0.00)
	No	58 (95.08)	27 (90.00)	31 (100.00)
Recurrence	Yes	7 (11.48)	5 (16.67)	2 (6.45)
	No	54 (88.52)	25 (83.33)	29 (93.55)
Type of work 1. Not employed 2. Light physical labor 3. Heavy physical labor 4. Child	1	27 (44.26)	1 (3.33)	26 (83.87)
	2	27 (44.26)	27 (90.00)	0 (0.00)
	3	2 (3.28)	2 (6.67)	0 (0.00)
	4	5 (8.20)	0 (0.00)	5 (16.13)
Previous treatments	No	54 (88.52)	24 (80.00)	30 (96.77)
	Steroid injection	4 (6.56)	4 (13.33)	0 (0.00)
	Physiotherapy	3 (4.92)	2 (6.67)	1 (3.23)
Trauma history	Yes	1 (1.64)	0 (0.00)	1 (3.23)
	No	60 (98.36)	30 (100.00)	30 (96.77)
Dominant Hand	Right	56 (91.80)	26 (86.67)	30 (96.77)
	Left	5 (8.20)	4 (13.33)	1 (3.23)
Using of immune suppressive drug	Yes	1 (1.64)	0 (0.00)	1 (3.23)
	No	60 (98.36)	30 (100.00)	30 (96.77)
Requirement for physical therapy (Postoperative)	Yes	4 (6.56)	2 (6.67)	2 (6.45)
	No	57 (93.44)	28 (93.33)	29 (93.55)
Complication	Yes	9 (14.75)	6 (20.00)	3 (9.68)
	No	52 (85.25)	24 (80.00)	28 (90.32)
Satisfaction	Excellent	51 (83.6)	23 (76.7)	28 (90.3)
	Moderate	7 (11.5)	5 (16.7)	2 (6.5)
	Poor	3 (4.9)	2 (6.6)	1 (3.2)

After administering local or general anesthesia and prophylactic antibiotics, an incision was made directly over the A1 pulley following the style of Bruner. Subsequently, the A1 pulley was incised after exploring and preserving adjacent neurovascular structures. Intraoperative traction on the flexor tendon proximal to the A1 pulley followed the incision. Before final closure, confirmation of the absence of triggering was assessed by ensuring full passive motion through passive digital extension and flexion, as well as traction of the flexor tendons proximal to the A1 pulley. Patient selection was based on a thorough search of the Department of Orthopedic Surgery archive for individuals undergoing surgical release for trigger fingers. This retrospective study received approval from the Institutional Review Board of our University. Patients exhibiting persistent trigger and/or fixed flexion contractures of the involved finger(s) resulting in functional limitations despite observation, therapy, and splinting were considered candidates for surgery. Patients under 18 years of age have been considered pediatric patients. Pediatric and adult patients who did not come for follow-up regularly and whose post-operative follow-up was less than three months were not included in the study. A standardized surgical technique was uniformly applied to all cases. The medical records of all 61 patients were meticulously reviewed. The study considered several demographic and clinical factors, including age, gender, dominant hand, body mass index, occupation, history of trauma, single or multiple finger involvement, and staging according to Green classification (Grade 1: Pain/history of catching, Grade 2: Demonstrable catching, but can actively extend the digit, Grade 3: Demonstrable locking, requiring passive extension, Grade 4: Fixed flexion contracture), presence of additional diseases, diabetes mellitus, recurrence, revision surgery, complication (superficial wound infection, superficial wound necrosis, scar sensitivity), utilization of non-surgical treatment methods (observation, injection, physical therapy), need for rehabilitation after surgery, time to return to work (day), the time interval from clinic initiation to the surgery (month), satisfaction (good, moderate, poor) and the duration of the follow-up period (month). The presence of comorbidities such as hypertension, asthma, coronary artery disease, renal failure, thyroid diseases, and rheumatological diseases were evaluated. In addition, the quick version of the disabilities of the arm, shoulder, and hand (QDASH); and the visual analog scale (VAS) were used to assess patients’ data.

Statistical analysis

The qualitative data among the variables in the study were summarized using counts and percentages. The normal distribution of quantitative data was assessed through the Shapiro-Wilk test. As the quantitative data did not exhibit a normal distribution, they were summarized using the median (minimum-maximum). Statistical analyses employed the Pearson chi-square test, Fisher's exact chi-square test, Mann-Whitney U test, and Kruskal-Wallis test where appropriate. Relationships between variables were examined using the Spearman correlation coefficient. A level of p < 0.05 was considered statistically significant in all applied statistical analyses. All analyses were conducted using SPSS (IBM Corp. Released 2019. IBM SPSS Statistics for Windows, Version 26.0. Armonk, NY: IBM Corp).

## Results

Of the 61 patients, 31 (50.8%) were children, 30 (49.2%) were adults, and a total of 91 fingers were evaluated in the study. Distribution of trigger fingers: 52 (57.1%) thumbs, six (6.5%) second fingers, 18 (19.7%) third fingers, 13 (14.2%) fourth fingers and two (2.1%) fifth fingers. The thumb was the most frequently affected finger in all cases. Thirty-five patients (57.3%) had a trigger finger on only one finger, while 26 patients (42.7%) had a trigger finger on more than one finger in all cases (Table 1). We found no statistically significant relationship between multiple-finger involvement and single-finger involvement in terms of complications, recurrence, and satisfaction in both pediatric and adult patients. 39 (63.9%) of the patients were female and 22 (36.1%) were male. There was no statistically significant relationship between male and female patients in terms of complications, recurrence, and satisfaction in both pediatric and adult patients. The average age of children was six, the average age of adults was 53, and the general average age was 29. The average follow-up period of pediatric patients was 49.77±27.56 months, the average follow-up period of adult patients was 38±24.75 months, and the average follow-up period of all patients was 43.98±26.66 months. Although a statistically significant difference was determined between pediatric and adult patients in terms of QDASH (p=0.018) and VAS (p=0.001) scores, there was no statistically significant relationship in terms of complications (p=0.301), recurrence (p=0.255) and satisfaction (p=0.283). The right hand was the dominant side for 56 patients (91.8%), while the left hand was dominant for five patients (8.2%). There was no statistically significant relationship between the dominant side and complications, recurrence, satisfaction, QDASH, and VAS scores in both pediatric and adult patients. Twelve patients (19.6%) had a positive history of diabetes mellitus. Pediatric patients had no positive history of diabetes mellitus. Twenty adult patients (67%) and three pediatric patients (9.7%) had comorbidities other than trigger finger without diabetes mellitus. Patients with diabetes were compared to those without diabetes in terms of QDASH, VAS score, complications, recurrence, and satisfaction. No statistically significant relationship was observed (Tables 2, 3, 4).

**Table 2:** Evaluation of prognostic factors in terms of complications in adult patients * Pearson chi-square test; **: Fisher’s exact chi-square test; p indicates the significance level

VARIABLES		COMPLICATION		p
		Yes n (%)	No n (%)	
Gender	Male	1 (8.33)	11 (91.67)	0.358^**^
	Female	5 (27.78)	13 (72.22)	
Number of affected fingers	Single	1 (6.67)	14 (93.33)	0.169^**^
	Multiple	5 (33.33)	10 (66.67)	
Grade- Green	2	2 (50.00)	2 (50.00)	<0.001^*^
	3	1 (4.55)	21 (95.45)	
	4	3 (100.00)	0 (0.00)	
Comorbidity	Yes	6 (30.00)	14 (70.00)	0.074^**^
	No	0 (0.00)	10 (100.00)	
Diabetes	Yes	2 (16.67)	10 (83.33)	1.0^**^
	No	4 (22.22)	14 (77.78)	
Additional operation	Yes	1 (33.33)	2 (66.67)	0.501^**^
	No	5 (18.52)	22 (81.48)	
Type of work 1. Not employed 2. Light physical labor 3. Heavy physical labor	1	0 (0.00)	1 (100.00)	0.659^*^
	2	6 (22.22)	21 (77.78)	
	3	0 (0.00)	2 (100.00)	
Previous treatments	No	5 (20.83)	19 (79.17)	0.751^*^
	Steroid injection	1 (25.00)	3 (75.00)	
	Physiotherapy	0 (0.00)	2 (100.00)	
Trauma history	Yes	0 (0.00)	0 (0.00)	-
	No	6 (20.00)	24 (80.00)	
Dominant Hand	Right	5 (19.23)	21 (80.77)	1.0^**^
	Left	1 (25.00)	3 (75.00)	
Using of immune suppressive drug	Yes	0 (0.00)	0 (0.00)	-
	No	6 (20.00)	24 (80.00)	
Requirement for physical therapy (Postoperative)	Yes	0 (0.00)	2 (100.00)	1.0^**^
	No	6 (21.43)	22 (78.57)	

**Table 3:** Evaluation of prognostic factors in terms of recurrence in adult patients * Pearson chi-square test; **: Fisher’s exact chi-square test; p indicates the significance level

VARIABLES		RECURRENCE		p
		Yes n (%)	No n (%)	
Gender	Male	1 (8.33)	11 (91.67)	0.622^**^
	Female	4 (22.22)	14 (77.78)	
Number of affected fingers	Single	1 (6.67)	14 (93.33)	0.330^**^
	Multiple	4 (26.67)	11 (73.33)	
Grade- Green	2	2 (50.00)	2 (50.00)	<0.001^*^
	3	0 (0.00)	22 (100.00)	
	4	3 (100.00)	0 (0.00)	
Comorbidity	Yes	5 (25.00)	15 (75.00)	0.140^**^
	No	0 (0.00)	10 (100.00)	
Diabetes	Yes	2 (16.67)	10 (83.33)	1.0^**^
	No	3 (16.67)	15 (83.33)	
Additional operation	Yes	1 (33.33)	2 (66.67)	0.433^**^
	No	4 (14.81)	23 (85.19)	
Type of work 1. Not employed 2. Light physical labor 3. Heavy physical labor	1	0 (0.00)	1 (100.00)	0.717^*^
	2	5 (18.52)	22 (81.48)	
	3	0 (0.00)	2 (100.00)	
Previous treatments	No	4 (16.67)	20 (83.33)	0.741*
	Steroid injection	1 (25.00)	3 (75.00)	
	Physiotherapy	0 (0.00)	2 (100.00)	
Trauma history	Yes	0 (0.00)	0 (0.00)	-
	No	5 (16.67)	25 (83.33)	
Dominant Hand	Right	4 (15.38)	22 (84.62)	0.538^**^
	Left	1 (25.00)	3 (75.00)	
Using of immune suppressive drug	Yes	0 (0.00)	0 (0.00)	-
	No	5 (16.67)	25 (83.33)	
Requirement for physical therapy (Postoperative)	Yes	0 (0.00)	2 (100.00)	1.0^**^
	No	5 (17.86)	23 (82.14)	

**Table 4:** Evaluation of prognostic factors in terms of satisfaction in adult patients * Pearson chi-square test; p indicates the significance level

VARIABLES		PATIENT SATISFACTION			p^*^
		Excellent n (%)	Moderate n (%)	Poor n (%)	
Gender	Male	10 (83.33)	2 (16.67)	0 (0.00)	0.485
	Female	13 (72.22)	3 (16.67)	2 (11.11)	
Number of affected fingers	Single	13 (86.67)	2 (13.33)	0 (0.00)	0.274
	Multiple	10 (66.67)	3 (20.00)	2 (13.33)	
Grade- Green	2	2 (50.00)	1 (25.00)	1 (25.00)	0.001
	3	21 (95.45)	1 (4.55)	0 (0.00)	
	4	0 (0.00)	2 (66.67)	1 (33.33)	
Comorbidity	Yes	15 (75.00)	3 (15.00)	2 (10.00)	0.571
	No	8 (80.00)	2 (20.00)	0 (0.00)	
Diabetes	Yes	10 (83.33)	2 (16.67)	0 (0.00)	0.485
	No	13 (72.22)	3 (16.67)	2 (11.11)	
Additional operation	Yes	2 (66.67)	1 (33.33)	0 (0.00)	0.663
	No	21 (77.78)	4 (14.81)	2 (7.41)	
Type of work 1. Not employed 2. Light physical labor 3. Heavy physical labor	1	1 (100.00)	0 (0.00)	0 (0.00)	0.908
	2	20 (74.07)	5 (18.52)	2 (7.41)	
	3	2 (100.00)	0 (0.00)	0 (0.00)	
Previous treatments	No	19 (79.17)	3 (12.50)	2 (8.33)	0.369
	Steroid injection	2 (50.00)	2 (50.00)	0 (0.00)	
	Physiotherapy	2 (100.00)	0 (0.00)	0 (0.00)	
Trauma history	Yes	0 (0.00)	0 (0.00)	0 (0.00)	-
	No	23 (76.67)	5 (16.67)	2 (6.67)	
Dominant Hand	Right	20 (76.92)	4 (15.38)	2 (7.69)	0.778
	Left	3 (75.00)	1 (25.00)	0 (0.00)	
Using of immune suppressive drug	Yes	0 (0.00)	0 (0.00)	0 (0.00)	-
	No	23 (76.67)	5 (16.67)	2 (6.67)	
Requirement for physical therapy (Postoperative)	Yes	2 (100.00)	0 (0.00)	0 (0.00)	0.772
	No	21 (75.00)	5 (17.86)	2 (7.14)	

Similarly, when comparing patients with and without comorbidities, there were no statistically significant differences in terms of QDASH and VAS scores, complications, recurrence, and satisfaction in both pediatric and adult patients. All patients were classified according to Green's staging and there were Grade 1: one patient (1.6%), Grade 2: six patients (9.9%), Grade 3: 49 patients (80.3%), Grade 4: five patients (8.2%). Recurrence occurred in seven (11.5%) patients, two of whom were children (6.4%) and five of whom were adults (16.6%). There was no statistically significant relationship between pediatrics and adults in terms of recurrence (p=0.255). The relationship between the increasing grade of the Green stage and the surgical recurrence was found to be statistically significant in adult patients (p<0.001) (Table 3). Simultaneously, a statistically significant relationship was found between the increasing grade in Green's classification and both complication rate (p<0.001) and lower satisfaction (p<0.001) in adult patients (Table 2, 4). However, no statistically significant relationship was identified between Green's classification and complications (p=0.129), recurrence (p=0.854), and satisfaction (p=0.143) in pediatric patients (Tables 5, 6, 7).

**Table 5:** Evaluation of prognostic factors in terms of complications in pediatric patients * Pearson chi-square test; **: Fisher’s exact chi-square test; p indicates the significance level

VARIABLES		COMPLICATION		p
		Yes n (%)	No n (%)	
Gender	Male	3 (30.00)	7 (70.00)	0.097^**^
	Female	0 (0.00)	21 (100.00)	
Number of affected fingers	Single	3 (15.00)	17 (85.00)	0.535^**^
	Multiple	0 (0.00)	11 (100.00)	
Grade- Green	2	0 (0.00)	2 (100.00)	0.129^*^
	3	2 (7.41)	25 (92.59)	
	4	1 (50.00)	1 (50.00)	
Comorbidity	Yes	1 (33.33)	2 (66.67)	0.271^**^
	No	2 (7.14)	26 (92.86)	
Diabetes	Yes	0 (0.00)	0 (0.00)	-
	No	3 (9.68)	28 (90.32)	
Additional operation	Yes	0 (0.00)	0 (0.00)	-
	No	3 (9.68)	28 (90.32)	
Previous treatments	No	2 (6.67)	28 (93.33)	0.097^**^
	Physiotherapy	1 (100.00)	0 (0.00)	
Trauma history	Yes	0 (0.00)	1 (100.00)	1.0^**^
	No	3 (10.00)	27 (90.00)	
Dominant Hand	Right	3 (10.00)	27 (90.00)	1.0^**^
	Left	0 (0.00)	1 (100.00)	
Using of immune suppressive drug	Yes	0 (0.00)	1 (100.00)	1.0^**^
	No	3 (10.00)	27 (90.00)	
Requirement for physical therapy (Postoperative)	Yes	1 (50.00)	1 (50.00)	0.187^**^
	No	2 (6.90)	27 (93.10)	

**Table 6:** Evaluation of prognostic factors in terms of recurrence in pediatric patients * Pearson chi-square test; **: Fisher’s exact chi-square test; p indicates the significance level

VARIABLES		RECURRENCE		p
		Yes n (%)	No n (%)	
Gender	Male	1 (10.00)	9 (90.00)	1.0^**^
	Female	1 (4.76)	20 (95.24)	
Number of affected fingers	Single	1 (5.00)	19 (95.00)	1.0^**^
	Multiple	1 (9.09)	10 (90.91)	
Grade- Green	2	0 (0.00)	2 (100.00)	0.854^*^
	3	2 (7.41)	25 (92.59)	
	4	0 (0.00)	2 (100.00)	
Comorbidity	Yes	0 (0.00)	3 (100.00)	1.0^**^
	No	2 (7.14)	26 (92.86)	
Diabetes	Yes	0 (0.00)	0 (0.00)	-
	No	2 (6.45)	29 (93.55)	
Additional operation	Yes	0 (0.00)	0 (0.00)	-
	No	2 (6.45)	29 (93.55)	
Previous treatments	No	2 (6.67)	28 (93.33)	1.0^**^
	Physiotherapy	0 (0.00)	1 (100.00)	
Trauma history	Yes	0 (0.00)	1 (100.00)	1.0^**^
	No	2 (6.67)	28 (93.33)	
Dominant Hand	Right	2 (6.67)	28 (93.33)	1.0^**^
	Left	0 (0.00)	1 (100.00)	
Using of immune suppressive drug	Yes	0 (0.00)	1 (100.00)	1.0^**^
	No	2 (6.67)	28 (93.33)	
Requirement for physical therapy (Postoperative)	Yes	0 (0.00)	2 (100.00)	1.0^**^
	No	2 (6.90)	27 (93.10)	

**Table 7:** Evaluation of prognostic factors in terms of patient satisfaction in pediatric patients *: Pearson chi-square test; p indicates the significance level

VARIABLES		PATIENT SATISFACTION			p^*^
		Excellent n (%)	Moderate n (%)	Poor n (%)	
Gender	Male	8 (80.00)	1 (10.00)	1 (10.00)	0.278
	Female	20 (95.24)	1 (4.76)	0 (0.00)	
Number of affected fingers	Single	18 (90.00)	1 (5.00)	1 (5.00)	0.693
	Multiple	10 (90.91)	1 (9.09)	0 (0.00)	
Grade- Green	2	2 (100.00)	0 (0.00)	0 (0.00)	0.143
	3	25 (92.59)	1 (3.70)	1 (3.70)	
	4	1 (50.00)	1 (50.00)	0 (0.00)	
Comorbidity	Yes	2 (66.67)	1 (33.33)	0 (0.00)	0.133
	No	26 (92.86)	1 (3.57)	1 (3.57)	
Diabetes	Yes	0 (0.00)	0 (0.00)	0 (0.00)	-
	No	28 (90.32)	2 (6.45)	1 (3.23)	
Additional operation	Yes	0 (0.00)	0 (0.00)	0 (0.00)	-
	No	28 (90.32)	2 (6.45)	1 (3.23)	
Previous treatments	No	27 (90.00)	2 (6.67)	1 (3.33)	0.946
	Physiotherapy	1 (100.00)	0 (0.00)	0 (0.00)	
Trauma history	Yes	1 (100.00)	0 (0.00)	0 (0.00)	0.946
	No	27 (90.00)	2 (6.67)	1 (3.33)	
Dominant Hand	Right	27 (90.00)	2 (6.67)	1 (3.33)	0.946
	Left	1 (100.00)	0 (0.00)	0 (0.00)	
Using of immune suppressive drug	Yes	1 (100.00)	0 (0.00)	0 (0.00)	0.946
	No	27 (90.00)	2 (6.67)	1 (3.33)	
Requirement for physical therapy (Postoperative)	Yes	1 (50.00)	1 (50.00)	0 (0.00)	0.034
	No	27 (93.10)	1 (3.45)	1 (3.45)	

There was a statistically significant difference between the increase in Green classification grade and both QDASH (p<0.001) and VAS (p<0.001) scores in all patients. The time interval from clinic initiation to surgery was 4±6.09 months for pediatric patients and 12.4±15.14 months for adults. Although there was a statistically significant relationship between the time interval from clinic initiation to the surgery and complications (p=0.033) in adult patients (Table 8), no statistically significant relationships were observed in terms of recurrence or satisfaction (Tables 8, 9). On the other hand, there was no statistically significant relationship between the time interval from clinic initiation to the surgery and complications, recurrence, and satisfaction in pediatric patients (Table 8, 9).

**Table 8:** Complication and recurrence in pediatric and adult patients Data is given as median (minimum-maximum); *: Mann Whitney U test; p indicates the significance level

COMPLICATION						
	Adult Patients		p^*^	Pediatric Patients		p^*^
VARIABLES	Yes [Median (Min-Max)]	No [Median (Min-Max)]		Yes [Median (Min-Max)]	No [Median (Min-Max)]	
Body mass index	26.83(20-33.29)	27.73(19.23-36)	0.568	20(16.52-23.87)	17.245(11-31)	0.349
Return to work	14.5(14-30)	14.5(3-30)	0.652	30(14-90)	14(3-30)	0.044
Duration to surgery	15.5(6-72)	8(0-48)	0.033	3(1-12)	1.5(1-24)	0.372
Follow up	37.5(13-74)	36(3-80)	0.716	30(25-47)	46.5(8-104)	0.367
RECURRENCE						
	Adult Patients			Pediatric Patients		
VARIABLES	Yes [Median (Min-Max)]	No [Median (Min-Max)]	p^*^	Yes [Median (Min-Max)]	No [Median (Min-Max)]	p^*^
Body mass index	26.12(20-30.85)	27.73(19.23-36)	0.191	24.18(17.35-31)	17.15(11-25)	0.198
Return to work	15(14-30)	14(3-30)	0.459	8.5(3-14)	14(3-90)	0.172
Duration to surgery	12(6-72)	12(0-48)	0.103	1(1-1)	2(1-24)	0.169
Follow up	38(13-74)	36(3-80)	0.717	71.5(39-104)	46(8-99)	0.376

**Table 9:** Satisfaction in pediatric and adult patients Data is given as median (minimum-maximum). *: Kruskall Wallis test; p indicates the significance level

VARIABLES	SATISFACTION							
	Adult			p^*^	Pediatric			p^*^
	Excellent	Good	Poor		Excellent	Good	Poor	
Body mass index	27.73 (19.23-36)	27.54(20-29.74)	25.71(20.56-30.85)	0.736	17.08(11-25)	25.5(20-31)	23.87(23.87-23.87)	0.120
Return to normal	14 (3-30)	30(15-30)	14(14-14)	0.029	14(3-30)	46.5(3-90)	30(30-30)	0.071
Duration to surgery	4(0-48)	12(12-19)	39(6-72)	0.198	1.5(1-24)	2(1-3)	12(12-12)	0.141
Follow up	37(3-80)	30(10-74)	32(13-51)	0.938	46(8-99)	64.5(25-104)	47(47-47)	0.858

## Discussion

Adult patients with advanced Green classification were found to have a negative impact on satisfaction due to high rates of complications and recurrence. Delayed surgical time was found to result in increased complications. However, it did not influence patients' satisfaction in long-term results. These two factors are the primary negative prognostic indicators for adult trigger finger surgery in our study. Koopman et al. investigated 2681 patients with trigger finger in terms of clinical characteristics associated with postoperative pain and hand function. They exhibited that the duration of symptoms in months and flexion contracture (advanced Green grade) were related to a poor clinical outcome three months postoperatively [12]. However, we found that the duration of symptoms did not significantly impact clinical outcomes, with an average follow-up of 38 months. Furthermore, in another study involving 3428 patients, Koopman et al. identified advanced-stage trigger finger and the time interval from clinic initiation to surgery as significant prognostic factors [13]. In addition to this study, the most important emphasis in our study is that the prognostic factors identified are in adult patients, and do not impact the clinical outcomes of pediatric patients. Beak et al. conducted a review of 71 cases of pediatric trigger thumb and observed that 63% of cases resolved spontaneously without intervention. However, the median time for regression was reported to be 48 months [14]. Giugale et al. suggested that the influence of factors such as disease severity, age, or other variables on the likelihood of symptom resolution remains unclear [10]. Marek et al. published that the age of the child at the time of surgery was an important factor and children who had surgery after the age of three years took many months to correct the contracture [15]. In our investigation, we observed that the duration until surgical intervention has no remarkable effect on the pediatric patient's prognosis. Hence, we recommend the implementation of an appropriate strategy for managing the waiting period associated with spontaneous regression in instances of this nature. To the best of our knowledge, the evaluation of prognostic factors of trigger finger surgery in terms of age is limited in the literature. Koopman et al. emphasized that the dominant side was a prognostic factor for complications [13]. However, in our study, although trigger finger was more common in the dominant hand, we did not find a significant relationship between dominant and non-dominant hands in terms of complications, recurrence, and patient satisfaction in both pediatric and adult patients. Concomitantly, we appraised the number of finger involvement as well as the dominant hand from a prognostic perspective. Cakmak et al. reported that patients with multiple-digit release experienced more pain and swelling in the acute stage compared to those with single-digit release. However, multiple-digit involvement did not lead to an increased limitation of range of motion (ROM) or a prolonged convalescence [16]. In our study, we established that multiple-digit involvement was not of prognostic value in both pediatric and adult patients. In a retrospective study conducted by Federer et al., diabetic patients were found to have a significantly higher rate of complications following trigger finger release compared to non-diabetic patients [17]. However, a prospective study by Stirling et al., which included 192 patients followed up for an average of 12 months postoperatively, revealed no differences in clinical score, complication rate, or patient satisfaction between diabetic and non-diabetic patients [18]. In our study, consistent with Stirling et al., we found no differences in clinical score, complication rate, recurrence, and patient satisfaction when comparing patients with diabetes to those without diabetes. In our study, unlike the other two studies, we observed that patients with comorbidities did not exhibit worse clinical scores or higher complication rates compared to those without comorbidities. While comorbidity is commonly observed in adults, Fahey et al.'s study indicates that pediatric and adult trigger finger surgery have outcomes similar to clinical results [19,20]. Consistent with this, our study found no significant differences between pediatric and adult patients in terms of complication, recurrence, and patient satisfaction. The limitation of our study is that it was retrospective research and did not include preoperative QDASH and VAS scores in the evaluation. With the prospective investigations, researchers could explore additional variables influencing postoperative patient outcomes.

## Conclusions

In conclusion, our study demonstrates that favorable outcomes can be achieved through well-planned surgical treatment in terms of prognostic criteria following accurate diagnostic evaluation. Future controlled prospective studies could refine patient treatment strategies, aiding clinical decision-making on optimal preoperative management and intervention timing to enhance outcomes after surgical A1 pulley release.

## References

[1] David M, Rangaraju M, Raine A (2017). Acquired triggering of the fingers and thumb in adults. BMJ.

[2] Gil JA, Hresko AM, Weiss AC (2020). Current concepts in the management of trigger finger in adults. J Am Acad Orthop Surg.

[3] Jeanmonod R, Harberger S, Waseem M (2023). Trigger finger. https://pubmed.ncbi.nlm.nih.gov/29083657/.

[4] Makkouk AH, Oetgen ME, Swigart CR, Dodds SD (2008). Trigger finger: etiology, evaluation, and treatment. Curr Rev Musculoskelet Med.

[5] McAuliffe JA (2010). Tendon disorders of the hand and wrist. J Hand Surg Am.

[6] Siddiqui AA, Rajput IM, Adeel M (2019). Outcome of percutaneous release for trigger digits in diabetic and non-diabetic patients. Cureus.

[7] Parmaksızoğlu F, Cansü E, Unal MB (2013). Trigger finger at the carpal tunnel level: three case reports. Acta Orthop Traumatol Turc.

[8] Koroglu M, Ertem K, Ergen E, Aslanturk O, Bram D (2015). Flexor Digitorum Profundus Injury in a. Newborn.Turk J Vasc Surg.

[9] Wong AL, Wong MJ, Parker R, Wheelock ME (2022). Presentation and aetiology of paediatric trigger finger: a systematic review. J Hand Surg Eur Vol.

[10] Dogru M, Erduran M, Narin S (2020). The effect of radial extracorporeal shock wave therapy in the treatment of trigger finger. Cureus.

[11] Çimen O, Nami Ş (2023). Does surgical experience affect the outcomes during percutaneous release of the trigger finger?. Cureus.

[12] Koopman JE, Hundepool CA, Wouters RM, Duraku LS, Smit JM, Selles RW, Zuidam JM (2022). Factors associated with self-reported pain and hand function following surgical A1 pulley release. J Hand Surg Eur Vol.

[13] Koopman JE, Zweedijk BE, Hundepool CA (2022). Prevalence and risk factors for postoperative complications following open A1 pulley release for a trigger finger or thumb. J Hand Surg Am.

[14] Baek GH, Kim JH, Chung MS, Kang SB, Lee YH, Gong HS (2008). The natural history of pediatric trigger thumb. J Bone Joint Surg Am.

[15] Marek DJ, Fitoussi F, Bohn DC, Van Heest AE (2011). Surgical release of the pediatric trigger thumb. J Hand Surg Am.

[16] Cakmak F, Wolf MB, Bruckner T, Hahn P, Unglaub F (2012). Follow-up investigation of open trigger digit release. Arch Orthop Trauma Surg.

[17] Federer AE, Baumgartner RE, Cunningham DJ, Mithani SK (2020). Increased rate of complications following trigger finger release in diabetic patients. Plast Reconstr Surg.

[18] Stirling PH, Jenkins PJ, Duckworth AD, Clement ND, McEachan JE (2020). Functional outcomes of trigger finger release in non-diabetic and diabetic patients. J Hand Surg Eur Vol.

[19] Fahey JJ, Bollinger JA (1954). Trigger-finger in adults and children. J Bone Joint Surg Am.

[20] Giugale JM, Fowler JR (2015). Trigger finger: adult and pediatric treatment strategies. Orthop Clin North Am.

